# Caffeine Treatment for Prostaglandin E1–Induced Apnea Prevention in Congenital Heart Disease Neonates: A Randomized Clinical Trial

**DOI:** 10.1155/ccrp/4923280

**Published:** 2025-05-11

**Authors:** Ladan Salamati, Bahar Dehghan, Mohammad Reza Sabri, Alireza Ahmadi, Mehdi Ghaderian, Chehreh Mahdavi, Davood Ramezani Nezhad, Atefeh Karbasi, Mohsen Sedighi

**Affiliations:** ^1^Pediatric Cardiovascular Research Center, Cardiovascular Research Institute, Isfahan University of Medical Sciences, Isfahan, Iran; ^2^Department of Pediatrics, Isfahan University of Medical Sciences, Isfahan, Iran; ^3^Trauma and Injury Research Center, Iran University of Medical Sciences, Tehran, Iran

**Keywords:** apnea, caffeine, congenital heart disease, neonates, prostaglandin

## Abstract

**Background:** Congenital heart diseases (CHDs) are structural abnormalities of the heart or great vessels. Prostaglandin E1 (PGE1) is used to maintain the ductus arteriosus open in neonates with ductal-dependent heart lesions but is associated with apnea. We aimed to investigate the effects of caffeine therapy on the occurrence of apnea in neonates with CHD.

**Methods:** This single-blinded randomized clinical trial was performed on 51 CHD neonates who were treated with PGE1 or PGE1 + caffeine. PGE1 dose ranged from 0.01 to 0.1 mcg/kg/min, and caffeine was administered initially at 20 mg/kg, followed by a daily bolus dose of 10 mg/kg. Demographic and clinical data, prevalence of apnea, and PGE1 side effects were recorded and analyzed.

**Results:** A total of 51 CHD neonates receiving PGE1 + caffeine (*n* = 25) and PGE1 (*n* = 26) were included. The median age of total neonates was 2 (1–7) days, and 57% were female. There was no statistically significant difference between the baseline characteristics of participants, but neonates in the caffeine group received a higher mean dose of PGE1 (0.03 ± 0.17 vs. 0.02 ± 0.02, *p*=0.049) over the course of the treatment. The prevalence of apnea was 20% in the PGE1 + caffeine group and 42% in the PGE1 group (*p*=0.086). In the Cox regression model, the age of neonates had a significant effect on time to apnea in patients receiving caffeine (HR = 0.87, *p*=0.04).

**Conclusion:** Our findings fail to demonstrate that caffeine therapy reduces PGE1-induced apnea. A larger randomized controlled trial is required to confirm or refute the efficacy of caffeine in reducing the incidence of apnea associated with PGE1 infusion.

**Trial Registration:** Iranian Registry of Clinical Trials: IRCT20220503054729N1

## 1. Introduction

Congenital heart diseases (CHDs) are structural abnormalities of the heart or intrathoracic great vessels occurring during fetal development and can be subdivided into acyanotic and cyanotic forms [1, 2]. CHD affects 8 to 9 per 1000 live births, around 25% of whom are cyanotic. It is estimated that 35% of deaths in infants with congenital malformations are related to cardiovascular anomalies [3, 4]. Prostaglandin E1 (PGE1) is used to maintain ductus arteriosus patency in infants having ductal-dependent congenital heart lesions with oxygen saturation of less than 75% until surgical intervention and has become an integral part of palliative therapy in pediatric cardiology. Nevertheless, apnea, respiratory depression, bradycardia, hypotension, hypokalemia, and fever are the most important adverse effects of PGE [5].

Apnea induced by infusion of PGE1 is reported in up to 12% of patients, proposing its effect on respiratory neural control [6]. Ductus arteriosus is uniquely sensitive to the vasodilatory effects of PG, which maintains ductal patency by acting on prostaglandin receptors, primarily EP4 [7]. In utero, both placenta and ductus arteriosus synthesize and release PGE2 which inhibits fetal breathing movements. Given the inhibitory effects of endogenous PGE on these movements, it is not surprising that exogenous PGE1 infusion can result in hypoventilation and apnea in the neonate and the need for mechanical ventilation [8]. Methylxanthines such as caffeine, theophylline, and aminophylline are extensively used for treating apnea of prematurity in preterm neonates [9]. Caffeine appears to function in apnea primarily as an adenosine receptor antagonist, but it displays some anti-inflammatory properties [10, 11]. Moreover, caffeine is reported to have a high therapeutic ratio, good enteral absorption, longer half-life, and fewer adverse effects. Therefore, its efficacy and tolerability make it the first choice of treatment for apnea [12, 13].

Given the depressant effects of PGE1 on breathing and the risk for increased respiratory instability, the current investigation was undertaken to explore if caffeine pretreatment could mitigate the apnea occurrence associated with PGE1 administration in CHD neonates.

## 2. Materials and Methods

### 2.1. Study Design and Patients

This single-blinded randomized clinical trial was performed on CHD neonates who were diagnosed postnatally and hospitalized in the neonatal intensive care unit (NICU) from August 2022 to April 2023. Inclusion criteria to enter the study were diagnosed CHD neonates who required PGE1 infusion (SpO_2_ ≤ 85%) and were hospitalized in the NICU. Diagnosis of the cardiac defects was made by an expert pediatric cardiologist. Neonates were excluded if they had prior initiation of PGE1 or were deemed to be at imminent risk of cardiovascular compromise. Also, neonates with noninvasive respiratory support, mechanical ventilation, concurrent infection, and sepsis were excluded from the study.

### 2.2. Sample Size

The primary endpoint of the study was comparing the occurrence of apnea between the study groups. The sample size estimation was based on the difference in the primary endpoint (decrease in the incidence of apnea) between the two study groups. According to the prior study by Lim et al. [14] which reported a statistically significant difference in the incidence of apnea in CHD neonates on PGE1 who received aminophylline or placebo (9.5% vs. 52.4%), with Type I error (5%), power 90%, and potential attrition rate of 5%, the estimated sample size was 25 for each group.

### 2.3. Randomization

A computer-based random number sequence was generated by a researcher who was not involved in the trial. The allocations were concealed from the investigators by using opaque sealed envelopes containing group allocations. Neonates who met our inclusion criteria were randomized in a 1:1 allocation to the PGE1 + caffeine and PGE1 groups, and the parents were blinded to their neonate's allocation group during the project period.

### 2.4. Treatment Protocol

Participants were allocated to groups of PGE1 (control) and PGE1 + caffeine (intervention). PGE1 was initiated in all neonates at a dose of 0.01 mcg/kg/min and increased gradually to 0.1 mcg/kg/min, if O_2_ saturation in neonates remained less than 75%. In the intervention group, the initial bolus dose was 20 mg/kg of caffeine citrate and the standard maintenance dose was 10 mg/kg/day of caffeine citrate. Whenever O_2_ saturation increased and/or neonates became ready to start oral feeding, caffeine administration continued through the oral route.

### 2.5. Patient Monitoring

Neonates were continuously monitored by cardiorespiratory telemetry until PGE1 was discontinued. Over the course of the treatment, patients were assessed for the maximum dose of prostaglandin infusion, the occurrence of apnea, the presence of respiratory distress, the need for intubation, and survival until discharge from the NICU. Apnea was defined as stopping breathing for at least 10 s with an associated decline in heart rate to < 100 bpm and oxygen saturation to < 80%. Neonates with major apneas who need positive pressure ventilation received noninvasive ventilation (NIV) or underwent mechanical ventilation if they did not respond to NIV. Apnea was diagnosed based on the subjective determination of respiratory cessation and bradycardia taken from the monitors and validated by the documentation of the attending neonatologist or qualified nurses. Neonates were also monitored for other side effects of PGE1 therapy such as hyperthermia, irritability, arrhythmias, or seizures as long as PGE1 was administered.

### 2.6. Statistical Analysis

Continuous data are expressed as mean (SD) or median (interquartile range [IQR]) values, and categorical data are expressed as frequency. To assess the normality of the data distribution, the Kolmogorov–Smirnov test was used. Based on the normality of data, Student's *t*-test or Mann–Whitney *U* test was used to compare continuous variables and the chi-square test with the Fisher exact test was applied to compare categorical variables. The Cox proportional hazards regression model with adjustment for neonate age, PGE1 dose, and gestational age was applied to investigate the association between time to apnea and caffeine therapy. The results were expressed as hazard ratios (HRs) and 95% confidence intervals (CI). Moreover, the Kaplan–Meier method was performed to compare time to apnea between two groups over the course of the caffeine treatment. Statistical analysis was conducted using the R software package (Version 4.3.1), and a *p* value less than 0.05 was considered statistically significant.

## 3. Results

**Figure 1:**
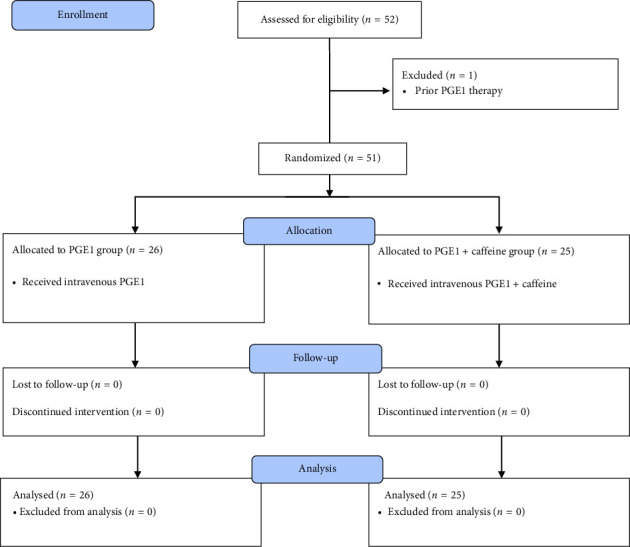
The flow diagram of the study.

Fifty-five CHD neonates were admitted to our center and hospitalized in the NICU. Of these, 52 children met our inclusion criteria and were enrolled in the study. As depicted in Figure 1, one neonate with a previous history of PGE1 administration was excluded from the study. The median age of the remaining 51 neonates was 2 (1–7) days and 57% were female (29 females versus 22 males).

Table 1 represents the comparison of baseline demographic and clinical characteristics of neonates at the time of admission to the NICU. There was no statistically significant difference between the characteristics of participants before randomization.  Table 2 summarizes and compares clinical outcomes and adverse effects attributed to pharmacotherapy in hospitalized neonates in the NICU. The mean dose of received PGE1 in the caffeine group over the course of the treatment was higher when compared to the control group (*p*=0.049). Prevalence of apnea in the caffeine + PGE1 group was relatively low compared with the PGE1 group but was not statistically significant (20% vs. 42%, *p*=0.086). Furthermore, caffeine therapy was not associated with a significant difference in the need for NIV or mechanical ventilation during treatment. There was no significant difference in the incidence of adverse events including fever, seizure, and tachycardia between groups.

**Figure 2:**
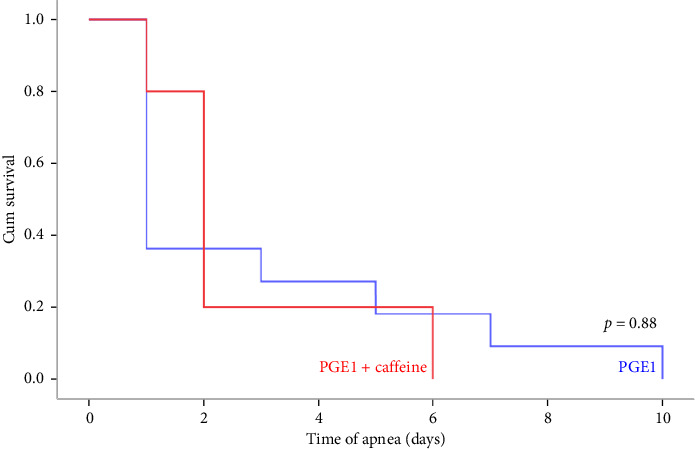
The Kaplan–Meier curves showing the probability of apnea in the neonates treated with and without caffeine over the course of the treatment in the NICU.

Table 3 describes the results of Cox regression analysis for the association between time to apnea and caffeine administration with adjustment for gestational age, PGE1 dose, and age of neonates. In the adjusted model, neonate age showed a significant effect on the time of apnea occurrence in neonates receiving caffeine. An increase in neonatal age decreased the risk of apnea occurrence (HR = 0.87, *p*=0.04), while caffeine administration did not have an effect according to the regression analysis (HR = 0.37, *p*=0.167).  Figure 2 shows the results of the Kaplan–Meier analysis of the apnea occurrence. The Kaplan–Meier curve was created according to the number of days before an event occurred (during Days 1–10) indicating a nonsignificant effect of caffeine therapy on the time to apnea event (*p*=0.88).

## 4. Discussion

It has been shown that PGE1 exerts adenosine-mediated effects on brainstem mechanisms of respiratory control, which may result in the destabilization of breathing in neonates being treated for CHD [15]. Therefore, caffeine could be a convenient treatment to prevent respiratory instability in neonates receiving PGE1 infusions. Caffeine's beneficial effect seems to be primarily mediated via a central blockade of adenosine A1 and A2A receptors, but it also has anti-inflammatory properties [10]. Therefore, neonates with CHD could benefit from caffeine as a noninvasive therapy to mitigate apnea and avoid adverse effects related to invasive ventilation [16]. The main objective of this randomized clinical trial was to explore the effects of caffeine administration on the apnea event in neonates with CHD receiving PGE1 in the NICU. Although the use of caffeine in neonates receiving PGE1 was associated with a partial reduction of apnea events (20% vs. 42%), it was not statistically significant when compared with the results of those neonates treated with PGE1 only.

Caffeine is one of the most commonly administrated drugs in the NICU and is a preferred drug for the treatment of apnea-related symptoms [17]. We observed a nonsignificant reduction in the occurrence of apnea in neonates receiving caffeine which is consistent with Higgins and Buck findings, reporting no difference in the incidence of apnea between children on low-dose alprostadil who received caffeine for prevention and those who did not [18]. Regarding adverse effects associated with caffeine, no noticeable events were seen except for two cases of tachycardia in neonates treated with caffeine. Also, the number of neonates who needed mechanical ventilation owing to apnea did not significantly differ between the two groups of trial. Carmo et al. suggested that infants with low-dose alprostadil treatment might not require mechanical ventilation and may benefit from a preventive dose of caffeine as an additional safeguard against the requirement of mechanical ventilation [19].

Neonates with various forms of CHD may depend on the patency of the duct to survive during the early neonatal period. Natural constriction of the ductus leads to low systemic cardiac output, shock, acidosis, and death [20]. PGE1 administration has allowed these neonates to survive until they can undergo surgical intervention. Nevertheless, PGE1 can cause apnea dose dependently in these vulnerable neonates, proposing an undesirable influence on the respiratory neural control system [21]. In our study, the Cox regression model indicated that increasing age of neonates decreased the prevalence of PGE1-induced apnea. Preterm infants have a delayed maturation of the respiratory system along with impaired brainstem-mediated homeostatic (respiratory and autonomic) control, predisposing them to apnea and hypoventilation that in turn may precipitate desaturation and/or bradycardia [22]. A relationship has been identified between PGE metabolites and apnea in preterm infants [23]. Moreover, apnea is found to be a dose-dependent side effect related to the use of PGE1 with a higher incidence in doses greater than 0.05 mcg/kg/min [24, 25]. However, dose dependence in the apnea occurrence as a side effect of PGE1 was not confirmed based on the results of regression analysis in our study. CHD neonates with caffeine treatment in our trial received a significantly increased dose of PGE1 during the trial, demonstrating that caffeine could be able to reverse the inhibitory effects of PGE1 on acute hypoxic ventilatory response and some indexes of respiratory instability [15].

The present study has some limitations. Our investigation is limited by the small sample size of the population which can affect the results of the study. Thus, further investigations with a multicenter design will likely be required to assess the efficacy of caffeine in preventing apnea in cyanotic CHD neonates. Also, we did not measure caffeine plasma levels in neonates, which could influence our results. Hence, measuring serum levels of PGE1 and caffeine in future investigations is proposed to validate caffeine effects more precisely.

## 5. Conclusion

The current study was designed to investigate the preventive effects of caffeine on PGE1-induced apnea in neonates with CHD. Our findings fail to demonstrate that caffeine therapy reduces PGE1-induced apnea. However, due to the small sample size, it is difficult to reach a definitive conclusion. Further randomized controlled trials with larger sample size and different dosages of caffeine are required to confirm or refute the efficacy of caffeine in reducing the incidence of apnea associated with PGE1 infusion.

## Figures and Tables

**Table 1:** Demographic and clinical data of neonates in two groups of the study.

Variables	PGE1 + caffeine (*n* = 25)	PGE1 (*n* = 26)	*p* value
Neonate age (days)^a^	3 (1–6.5)	2 (1–7.5)	0.969
Weight (kg)^a^	2.91 ± 0.54	3.05 ± 0.33	0.264
Sex (*n*)^b^			
Male	10 (40%)	12 (46%)	0.675
Female	15 (60%)	14 (54%)	
Gestational age (weeks)^a^	38 (37–38)	38 (37–38)	0.388
Cardiac diagnosis (*n*)^b^			
Hypoplastic left heart syndrome	2 (8%)	2 (8%)	
Transposition of the great vessels	10 (40%)	8 (31%)	
Coarctation of aorta	3 (12%)	3 (11.5%)	
Severe pulmonary stenosis	2 (8%)	2 (8%)	
Pulmonary atresia	4 (16%)	7 (27%)	0.340
Tetralogy of Fallot	1 (4%)	0	
Ebstein anomaly	1 (4%)	1 (4%)	
Tricuspid atresia	2 (8%)	0	
Atrioventricular septal defect	0	4 (15%)	
SBP (mm Hg)^a^	79.80 ± 5.05	78.56 ± 5.96	0.432
DBP (mm Hg)^a^	45.80 ± 6.42	46.04 ± 4.10	0.876
MBP (mm Hg)^a^	56.12 ± 5.64	58.36 ± 3.81	0.107
Heart rate (per minute)^a^	144.76 ± 12.01	139.80 ± 8.76	0.102
Respiratory rate (per minute)^a^	50.08 ± 4.32	47.48 ± 4.59	0.054
Body temperature (°C)^a^	36.84 ± 0.27	36.72 ± 0.21	0.106
FBS (mg/dL)^a^	108.08 ± 12.05	111.76 ± 9.76	0.241
Hemoglobin (g/dL)^a^	15.84 ± 1.37	15.76 ± 1.64	0.853
MCV (femtoliter)^a^	78.40 ± 2.34	77.84 ± 2.35	0.404
pH^a^	7.35 ± 0.02	7.34 ± 0.02	0.154
SpO_2_ (%)^a^	80 (79–83.5)	78 (77–81.5)	0.053
PaO_2_ (mm Hg)^a^	33.49 ± 1.17	33.41 ± 1.63	0.842
PaCO_2_ (mm Hg)^a^	43.24 ± 3.96	44.92 ± 3.61	0.124
HCO_3_ (mmol/L)^a^	24.80 ± 2.25	25.08 ± 1.73	0.624

Abbreviations: DBP = diastolic blood pressure, FBS = fasting blood sugar, MBP = mean blood pressure, MCV = mean corpuscular volume, SBP = systolic blood pressure.

^a^Continuous data are presented as mean (SD) or median (IQR).

^b^Categorical data are presented as frequency (%).

**Table 2:** Clinical outcomes of neonates over the course of the treatment.

Variables	PGE1 + caffeine (*n* = 25)	PGE1 (*n* = 26)	*p* value
Mean PGE1 dose (mcg/kg/min)^a^	0.03 ± 0.17	0.02 ± 0.02	0.049
Duration of PGE1 injection (days)^a^	7.75 ± 6.98	9.19 ± 7.48	0.486
Apnea^b^	5 (20%)	11 (42%)	0.086
Seizure^b^	1 (4%)	1 (4%)	0.745
Tachycardia^b^	2 (8%)	0	0.235
Fever^b^	0	1 (4%)	0.510
NIV^b^	4 (16%)	5 (19%)	0.762
Intubation^b^	8 (32%)	5 (19%)	0.235
Cardiac arrest^b^	0	1 (4%)	0.510
Mortality^b^	2 (8%)	2 (8%)	0.680

Abbreviation: NIV = noninvasive ventilation.

^a^Continuous data are presented as mean ± standard deviation.

^b^Categorical data are presented as frequency (%).

**Table 3:** Association between apnea time and caffeine therapy using the Cox proportional hazards model.

Variables	Unadjusted			Adjusted		
	HR	95% CI	*p* value	HR	95% CI	*p* value
Caffeine therapy	0.98	0.32–2.99	0.985	0.37	0.09–1.49	0.165
Gestational age				0.59	0.30–1.14	0.117
PGE1 dose				1.24	0.49–3.10	0.645
Neonate age				0.87	0.76–0.99	0.040

Abbreviations: CI = confidence interval, HR = hazard ratio.

## Data Availability

The data that support the findings of this study are available from the corresponding author upon reasonable request.

## References

[1] Desai K., Rabinowitz E. J., Epstein S. (2019). Physiologic Diagnosis of Congenital Heart Disease in Cyanotic Neonates. *Current Opinion in Pediatrics*.

[2] Bigdelian H., Montazeri M., Sedighi M., Mansouri M., Amanollahi A. (2023). Topical and Intravenous Tranexamic Acid in Acyanotic Children Undergoing Congenital Heart Surgery: A Randomized Clinical Trial. *Journal of Surgical Research*.

[3] Galvis M. M. O., Bhakta R. T., Tarmahomed A., Mendez M. D. (2023). *Cyanotic Heart Disease*.

[4] Segura T., Gatzoulis M. A. (2019). Where Are We with Coronary Artery Disease for the Cyanotic Patient with Congenital Heart Disease?. *International Journal of Cardiology*.

[5] Yokoyama U., Minamisawa S., Shioda A. (2014). Prostaglandin E2 Inhibits Elastogenesis in the Ductus Arteriosus via EP4 Signaling. *Circulation*.

[6] Vari D., Xiao W., Behere S., Spurrier E., Tsuda T., Baffa J. M. (2021). Low-dose Prostaglandin E1 Is Safe and Effective for Critical Congenital Heart Disease: Is it Time to Revisit the Dosing Guidelines?. *Cardiology in the Young*.

[7] Stoller J. Z., DeMauro S. B., Dagle J. M., Reese J. (2012). Current Perspectives on Pathobiology of the Ductus Arteriosus. *Journal of Clinical & Experimental Cardiology*.

[8] Huang F. K., Lin C. C., Huang T. C. (2013). Reappraisal of the Prostaglandin E1 Dose for Early Newborns with Patent Ductus Arteriosus-dependent Pulmonary Circulation. *Pediatrics & Neonatology*.

[9] Abu-Shaweesh J. M., Martin R. J. (2017). Caffeine Use in the Neonatal Intensive Care Unit. *Seminars in Fetal and Neonatal Medicine*.

[10] Köroğlu Ö A., MacFarlane P. M., Balan K. V. (2014). Anti-inflammatory Effect of Caffeine Is Associated with Improved Lung Function after Lipopolysaccharide-Induced Amnionitis. *Neonatology*.

[11] Ricciotti E., FitzGerald G. A. (2011). Prostaglandins and Inflammation. *Arteriosclerosis, Thrombosis, and Vascular Biology*.

[12] Nayak K., Nayak K., Lewis L. E. S., Kamath A., Purkayastha J. (2019). Acute Hemodynamic Effects of Methylxanthine Therapy in Preterm Neonates: Effect of Variations in Subgroups. *Journal of Tropical Pediatrics*.

[13] Aranda J. V., Beharry K. D. (2020). Pharmacokinetics, Pharmacodynamics and Metabolism of Caffeine in Newborns. *Seminars in Fetal and Neonatal Medicine*.

[14] Lim D., Kulik T. J., Kim D. W., Charpie J. R., Crowley D. C., Maher K. O. (2003). Aminophylline for the Prevention of Apnea during Prostaglandin E1 Infusion. *Pediatrics*.

[15] Mitchell L., Mayer C., Raffay T., DiFiore J., MacFarlane P. (2019). Caffeine Prevents Prostaglandin E1‐induced Disturbances in Respiratory Neural Control: Therapeutic Implications for Infants Treated for Congenital Heart Disease. *The FASEB Journal*.

[16] Henderson‐Smart D. J., De Paoli A. G. (2010). Methylxanthine Treatment for Apnoea in Preterm Infants. *Cochrane Database of Systematic Reviews*.

[17] Steer P., Flenady V., Shearman A. (2004). High Dose Caffeine Citrate for Extubation of Preterm Infants: a Randomised Controlled Trial. *Archives of Disease in Childhood-Fetal and Neonatal Edition*.

[18] Higgins K. L., Buck M. L. (2020). Caffeine Citrate for the Prevention of Apnea Associated with Alprostadil Infusions. *Journal of Pediatric Pharmacology and Therapeutics*.

[19] Carmo K. A., Barr P., West M., Hopper N. W., White J. P., Badawi N. (2007). Transporting Newborn Infants with Suspected Duct Dependent Congenital Heart Disease on Low-Dose Prostaglandin E1 without Routine Mechanical Ventilation. *Archives of Disease in Childhood-Fetal and Neonatal Edition*.

[20] Yun S. W. (2011). Congenital Heart Disease in the Newborn Requiring Early Intervention. *Korean journal of pediatrics*.

[21] Siljehav V., Hofstetter A. M., Leifsdottir K., Herlenius E. (2015). Prostaglandin E2 Mediates Cardiorespiratory Disturbances during Infection in Neonates. *The Journal of Pediatrics*.

[22] Moon R. Y., Horne R. S., Hauck F. R. (2007). Sudden Infant Death Syndrome. *The Lancet*.

[23] Hoch B., Bernhard M. (2000). Central Apnoea and Endogenous Prostaglandins in Neonates. *Acta Paediatrica*.

[24] Naiyananon F., Dissaneevate S., Thatrimontrichai A. (2024). Predictors of High Maintenance Prostaglandin E1 Doses in Neonates with Critical Congenital Heart Disease-ductal-dependent Pulmonary Circulation during Preoperative Care. *Pediatrics & Neonatology*.

[25] Mohammed S., Nour I., Shabaan A. E., Shouman B., Abdel-Hady H., Nasef N. (2015). High versus Low-Dose Caffeine for Apnea of Prematurity: a Randomized Controlled Trial. *European Journal of Pediatrics*.

